# Transcriptional analysis of the three Nlrp1 paralogs in mice

**DOI:** 10.1186/1471-2164-14-188

**Published:** 2013-03-18

**Authors:** Inka Sastalla, Devorah Crown, Seth L Masters, Andrew McKenzie, Stephen H Leppla, Mahtab Moayeri

**Affiliations:** 1 Microbial Pathogenesis Section, Laboratory of Parasitic Diseases, National Institute of Allergy and Infectious Diseases, National Institutes of Health, 33 North Drive, Bethesda, MD, 20892-3202, USA; 2 The Walter and Eliza Hall Institute of Medical Research, Inflammation Division, 1G Royal Parade, Parkville, Victoria, 3052, Australia

**Keywords:** Nlrp1, Inflammasome, Anthrax toxins, Lethal toxin, *Bacillus anthracis*, Splice variants

## Abstract

**Background:**

Signals of danger and damage in the cytosol of cells are sensed by NOD-like receptors (NLRs), which are components of multiprotein complexes called inflammasomes. Inflammasomes activate caspase-1, resulting in IL-1-beta and IL-18 secretion and an inflammatory response. To date, the only known activator of rodent Nlrp1 is anthrax lethal toxin (LT), a protease secreted by the bacterial pathogen *Bacillus anthracis.* Although susceptibility of mouse macrophages to LT has been genetically linked to Nlrp1b, mice harbor two additional *Nlrp1* paralogs in their genomes (*Nlrp1a* and *Nlrp1c)*. However, little is known about their expression profile and sequence in different mouse strains. Furthermore, simultaneous expression of these paralogs may lead to competitional binding of Nlrp1b interaction partners needed for inflammasome activation, thus influencing macrophages susceptibility to LT. To more completely understand the role(s) of *Nlrp1* paralogs in mice, we surveyed for their expression in a large set of LT-resistant and sensitive mouse macrophages. In addition, we provide sequence comparisons for Nlrp1a and report on previously unrecognized splice variants of *Nlrp1b*.

**Results:**

Our results show that macrophages from some inbred mouse strains simultaneously express different splice variants of *Nlrp1b*. In contrast to the highly polymorphic *Nlrp1b* splice variants, sequencing of expressed *Nlrp1a* showed the protein to be highly conserved across all mouse strains. We found that *Nlrp1a* was expressed only in toxin-resistant macrophages, with the sole exception of expression in LT-sensitive CAST/EiJ macrophages.

**Conclusions:**

Our data present a complex picture of Nlrp1 protein variations and provide a basis for elucidating their roles in murine macrophage function. Furthermore, the high conservation of Nlrp1a implies that it might be an important inflammasome sensor in mice.

## Background

The innate immune response is the initial line of defense against invading pathogens. The first responder cells of the immune system express proteins that have evolved to sense conserved microbial structures and to facilitate the induction of a protective inflammatory response. While extracellular microbial stimuli are sensed by surface-associated Toll-like receptors [1], recognition of intracellular pathogen and danger-associated molecular patterns (PAMPs and DAMPs) is mediated by cytosolic sensor proteins. These sensors, known as Nucleotide Oligomerization Domain (NOD)-like receptors (NLRs), can form multi-protein complexes called inflammasomes. Multiple domains have been recognized within these NLRs (for review, see [2]). The nucleotide-binding domain (NBD/NACHT) is central to all NLRs and is believed to oligomerize in an ATP-dependent fashion in response to specific stimuli, thus leading to NLR activation. The leucine-rich repeat (LRR) region is involved in signal sensing. In mouse Nlrp1b proteins, the FIIND (function to find) domain has recently been shown to undergo autoproteolysis which is required but not sufficient for the activation of this particular inflammasome by anthrax lethal toxin [3,4]. The C-terminal CARD (caspase recruitment) domain directly interacts with caspase-1. Many NLRs also contain a pyrin domain (N-terminally located in the case of human Nlrp1), with which they associate with the scaffold protein ASC (apoptosis-associated speck-like protein) [5]. Rodent Nlrp1 proteins, however, lack the N-terminal pyrin domain found in human Nlrp1, and instead harbor a region named NR100 [6], that has little homology to domains of other NLRs.

NLRs have been shown to be activated in response to a variety of stimuli including viral DNA, pore-forming toxins, proteases, bacterial flagellin, and crystalline matter such as alum or uric acid (for review see [2,7,8]). Activation of these sensors leads to inflammasome formation, recruitment of caspase-1, and its subsequent proteolytic activation. Activated caspase-1 then cleaves the proinflammatory cytokines IL-1β and IL-18, allowing for their secretion [2].

While some NLRs such as the well-characterized Nlrp3 are activated by a wide range of seemingly dissimilar signals [9], others, such as Nlrc4/NAIP5/NAIP6, gain their specificity due to activation through physical association with defined motifs in proteins like bacterial flagellin [10,11]. Similarly, anthrax lethal toxin (LT) is currently the only known activator of Nlrp1 in cells [6]. LT is comprised of the receptor-binding and channel-forming moiety protective antigen (PA) and lethal factor (LF), a zinc-dependent protease that cleaves most MAP kinase kinases [12,13]. While LT does not activate human Nlrp1, it activates certain rodent Nlrp1 proteins in macrophages, resulting in both the typical inflammatory response and the programmed cell death called pyroptosis [6,14]. In rats, the mechanism by which LT activates the Nlrp1 inflammasome has recently been clarified [15]. Sensitivity of rat macrophages to pyroptosis in response to LT is directly linked to the presence of certain short amino acid sequences at the most polymorphic site within the N-terminal NR100 domain. Nlrp1 from toxin-sensitive rat macrophages contains a sequence at this site that is cleaved by LF, resulting in toxin-mediated inflammasome activation [16]. Thus, the N-terminal region of rat Nlrp1 appears to be important to its biological function.

In mice, determining the role of Nlrp1 is made difficult because the genome contains three *Nlrp1* paralogs, *Nlrp1a, Nlrp1b,* and *Nlrp1c*, arranged in tandem on chromosome 11. In contrast to the Nlrp1c protein, which is truncated so as to lack the CARD domain, Nlrp1a and Nlrp1b contain all domains characteristic of murine NLRs. Using a genetic approach, Boyden and Dietrich [17] linked sensitivity to anthrax LT in mouse macrophages to Nlrp1b. They reported that *Nlrp1b* was the only paralog uniformly expressed in the LT-resistant and sensitive macrophages from all four inbred mouse strains examined, whereas *Nlrp1a* and *Nlrp1c* were not expressed in LT-sensitive macrophages of 129S1/SvlmJ mice, making the latter two paralogs unlikely candidates for the LT sensitivity locus. Furthermore, transgenic expression of an LT-sensitive Nlrp1b protein in LT-resistant macrophages sensitized them, consistent with other evidence that sensitivity is a dominant trait. Subsequently, after sequencing and comparing *Nlrp1b* from 18 inbred mice, Boyden and Dietrich inferred the existence of 5 different *Nlrp1b* alleles of which 3 are expressed by LT-resistant macrophages of 9 inbred strains. Two *Nlrp1b* alleles are associated with LT-sensitivity. Sequence comparisons showed most of the alleles to be very similar, with the exception of the allele expressed by C57BL6/J, A/J, and I/LnJ mice; its protein contains over 200 polymorphisms when compared to other alleles [17].

The existence of three paralogs in mice led us to consider whether the simultaneous expression of these proteins, which share over 70% protein sequence homology, might result in competition for the putative Nlrp1 binding partners needed for inflammasome activation. Thus, to more completely understand factors that may control sensitivity to LT-induced cell death, we surveyed the expression of all three paralogs in LT-resistant and sensitive macrophages derived from a large set of mouse strains. Furthermore, we provide sequence comparisons of the previously uncharacterized and highly conserved Nlrp1a protein, evidence for expression of *Nlrp1b* splice variants, and we survey tissue-wide expression of both *Nlrp1a* and *Nlrp1b*.

## Results

### Expression analysis and sequence comparison of *Nlrp1a* in anthrax lethal toxin-resistant and sensitive macrophages

**Figure 1:**
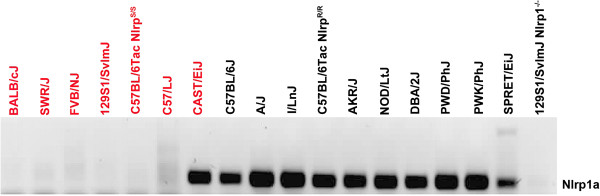
**Expression profile of *Nlrp1a.*** LT-resistant (black) or sensitive (red) macrophages of indicated mouse strains were PCR analyzed for expression of *Nlrp1a* using cDNA as template.

We analyzed expression of *Nlrp1a* from a large set of inbred mice using previously published primers [17]. Complimentary DNA from macrophages of 129S1 Nlrp1 null-mice (*Nlrp1*^−/−^) having all three paralogs deleted [18] served as a negative control. We found that *Nlrp1a* expression showed a near-perfect correlation between its expression and macrophage resistance to LT (Figure 1). All macrophages resistant to LT (denoted by black labels in Figure 1) expressed *Nlrp1a*. In contrast, macrophages sensitive to the toxin (red labels) did not express *Nlrp1a*, with the sole exception of CAST/EiJ macrophages. We also found *Nlrp1a* to be expressed in macrophages of C57BL/6Tac mice having an LT-resistant (*Nlrp*^R/R^) phenotype, but not in LT sensitive (*Nlrp*^S/S^) ones of the matching congenic mice, which maintain a small region of 129S1/SvlmJ-derived chromosome 11 including the *Nlrp1* locus (Figure 1) [19]. Analysis of *Nlrp1a* expression in 14 different tissues (bone marrow, spleen, liver, kidney, heart, lung, brain, stomach, muscle, spinal cord, thymus, adrenals, uterus, ovaries) also indicated that the gene was not expressed in Balb/cJ (Nlrp^S/S^) mice, but expressed in a majority of C57BL/6J (Nlrp^R/R^) tissues (Additional file 1: Table S1).

**Figure 2:**
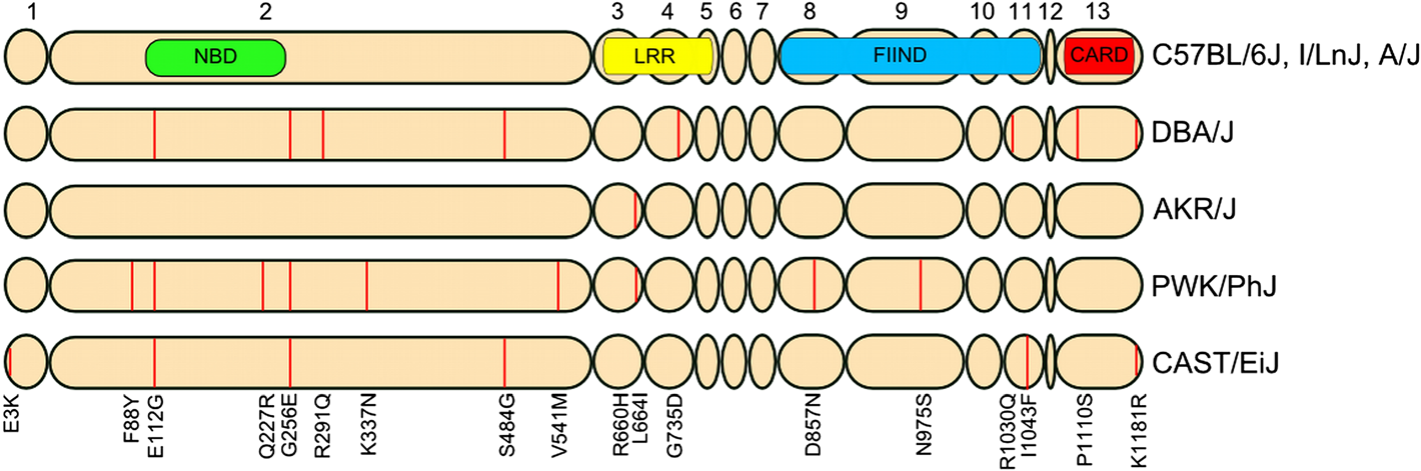
**Schematic overview of the five Nlrp1a variants identified in this study.** Exon numbers are given on the top, and vertical lines and amino acid substitutions in exon ovals indicate polymorphisms. Locations of domains were determined according to the annotated Nlrp1a protein sequence (GenBank CAM25466). NBD, nucleotide-binding domain; LRR, leucine-rich repeats; FIIND, function-to-find domain; CARD, caspase recruitment domain.

**Figure 3:**
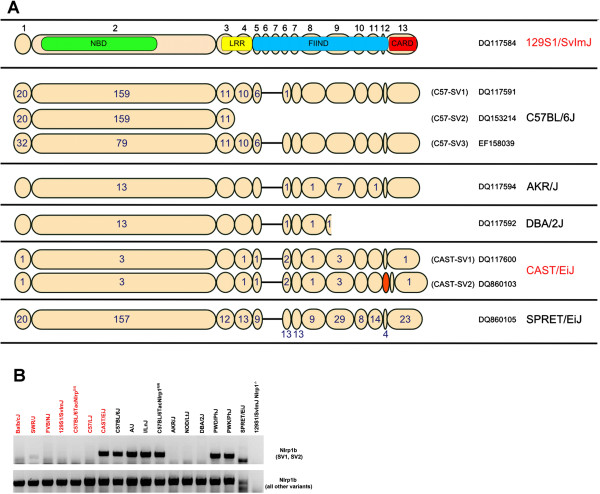
**(A) Schematic overview of the Nlrp1b (splice) variants deposited prior to this study (roughly to scale).** A representative mouse strain whose macrophages express a particular variant is shown on the right, and LT-sensitivity is indicated in red. The number of amino acid polymorphisms relative to the 129S1/SvImJ variant is indicated in (or below) the respective exons. Exon numbers are given on the top. *Nlrp1b* 129S1/SvImJ has exons 6 and 7 duplicated and transcribed, a unique feature not known to be present in other *Nlrp1b* genes. CAST/EiJ splice variant (SV) 2 contains an additional exon indicated in orange. GenBank accession numbers are shown on the right. NBD and other domain identifiers are as in Figure 2. Locations of domains were determined according to the annotated Nlrp1b C57-SV1 sequence. Schematic was adapted from [17]. (**B**) Expression profile of *Nlrp1b* splice variants. LT-resistant (black) or sensitive (red) macrophages of indicated mouse strains were PCR analyzed for expression of *Nlrp1b* using cDNA as template. The upper panel shows the expression profile of C57-SV1 and SV2 of *Nlrp1b*, the lower panel depicts the expression profile of all other variants.

Because of the interesting observation that CAST/EiJ macrophages were the only LT sensitive macrophages to express *Nlrp1a* (Figure 1), we wanted to exclude the possibility that *Nlrp1a* in this strain encodes for a truncated or possibly highly polymorphic protein, which would then be unable to compete for Nlrp1b binding partners, thus possibly resulting in LT-sensitivity in this particular strain only through absence of competition for Nlrp1b. Therefore, we sequenced (cDNA) *Nlrp1a* from LT-resistant (I/LnJ, A/J, PWK/PhJ, AKR/J, DBA/2J) and LT-sensitive (CAST/EiJ) macrophages. Figure 2 shows the five identified protein variants for Nlrp1a and the corresponding non-synonymous polymorphisms (indicated by vertical red lines). Comparison of the Nlrp1a protein sequences revealed that in striking contrast to Nlrp1b, which is highly polymorphic across different mouse strains (as per Figure 3A), Nlrp1a was highly conserved in all strains tested, paralleling the situation for the single expressed rat Nlrp1 paralog [15]. Macrophages with the most (nine) Nlrp1a polymorphisms were those of the PWK/PhJ mice, while the ones from AKR/J and CAST/EiJ showed one and six amino acid changes, respectively, compared to the C57BL/6J sequence (Figure 2). Interestingly, when comparing our *Nlrp1a* sequences to published C57BL/6J data, we discovered the presence of two deposited C57BL/6 transcript sequences in GenBank (GB) (AY355339 and DQ117601), which predict translated proteins of 1300 and 1182 amino acids, respectively (Additional file 2: Figure S1). The *Nlrp1a* macrophage-derived cDNA of the C57BL/6J mouse that we sequenced corresponded to that of the shorter protein and we used this sequence as reference for our alignment. Overall, the high degree of *Nlrp1a* sequence conservation among different mouse strains, in contrast to the immediately adjacent highly polymorphic *Nlrp1b* locus, suggests that this inflammasome sensor is likely to play an important role in sensing a yet unknown conserved danger signal.

### Expression of *Nlrp1b* and its splice variants

While analyzing *Nlrp1b* expression in inbred strains, we noted that for some mice, multiple splice variants (SV) of *Nlrp1b* were deposited in GB. Figure 3A gives an overview of all the different Nlrp1b variants, some unpublished, which were deposited previous to this work. Upon reviewing these deposited sequences and in light of our discovery described below, it became apparent that the initial description of Nlrp1b “alleles” is, at least to some degree, incorrect and misleading. For ease of reading and for accuracy, we will refer to “alleles” as protein “variants” or to transcripts as “splice variants”. We found that three *Nlrp1b* SVs were deposited for C57BL/6J macrophages. The translated C57-SV1 corresponds to the published, highly polymorphic C57BL/6J sequence of Nlrp1b (GB DQ117591) [17], while C57-SV2 (GB DQ153214) is a prematurely (after exon 3) truncated form of C57-SV1 (Figure 3A). In contrast, the translated sequence of C57-SV3 (GB EF158039) represents a protein that differs strikingly from the other two variants in the first 600 amino acids (encoded by exon 1 and 2), where all three share only 69% sequence identity. Interestingly, in these two exons, C57-SV3 is more similar to Nlrp1b variants of mice other than C57BL/6J (except SPRET/EiJ), sharing 82% identity. Of possible functional importance, this region of Nlrp1b encompasses the N-terminal do main of unknown function together with the NBD/NACHT domain needed for oligomerization and activation (Figure 3A). Furthermore, two CAST/EiJ sequences that differ by 24 amino acids in the FIIND domain (GB DQ1117600 and DQ860103, encoded by CAST-SV1 and CAST-SV2, respectively) were reported. The insertion in the latter sequence occurs between exons 11 and 12 (Figure 3A); thus, CAST-SV2 appears to incorporate an additional exon, which may impact FIIND domain-mediated activation of the translated protein [4]. We also discovered an *Nlrp1b* sequence derived from *Mus spretus* which resembles the C57-SV1 in the N-terminal region, but contains many polymorphisms in the C-terminal, usually conserved region of the translated Nlrp1b protein. This feature makes it different from most other Nlrp1b variants; thus, when referring to “all other Nlrp1b variants” in this manuscript, we exclude this unique SPRET/EiJ sequence.

To survey for the expression of different *Nlrp1b* (splice) variants in LT-resistant and sensitive macrophages, we designed two exon 2 specific primer sets that allowed us to distinguish the unique C57-SV1 and C57-SV2 transcripts from C57-SV3 and all other *Nlrp1b* variants. We found that indeed, LT-resistant macrophages of C57BL/6J and of all other mouse strains known to express the SV1 variant express at least two SVs (Figure 3B), and sequencing confirmed that they correspond to the deposited C57-SV1 and SV3 (Figure 3A). Interestingly, in macrophages of CAST/EiJ mice, our screen identified the expression of an additional SV (termed CAST-SV3, not presented in Figure 3), and subsequent sequence analysis of the amplicon showed it to be 99% identical to C57-SV1 (over a region of 456 nucleotides, spanning position 1351–1806 of the C57-SV1 sequence) (GB NM_001040696). These results show that mouse macrophages with opposing LT sensitivities (such as from CAST/EiJ and C57BL/6J mice) express at least two Nlrp1b proteins.

When we surveyed for expression of *Nlrp1b* SVs in fourteen other tissues (Additional file 1: Table S1), we found that in contrast to *Nlrp1a*, *Nlrp1b* was expressed in all tissues of BALB/cJ animals, except the stomach. For this particular tissue, it appeared that our primers cross-reacted with DNA from *Roseburia*, and it is possible that preferred sequence amplification led to a loss of *Nlrp1b*-specific PCR signals. Likewise, we found evidence of expression for *Nlrp1b* splice variants SV1/2 and SV3 in all tested C57BL/6J tissues, with the exception of the uterus, which lacked expression of SV1/2 (Additional file 1: Table S1).

### Genomic locations of identified *Nlrp1b* splice variants

**Figure 4:**
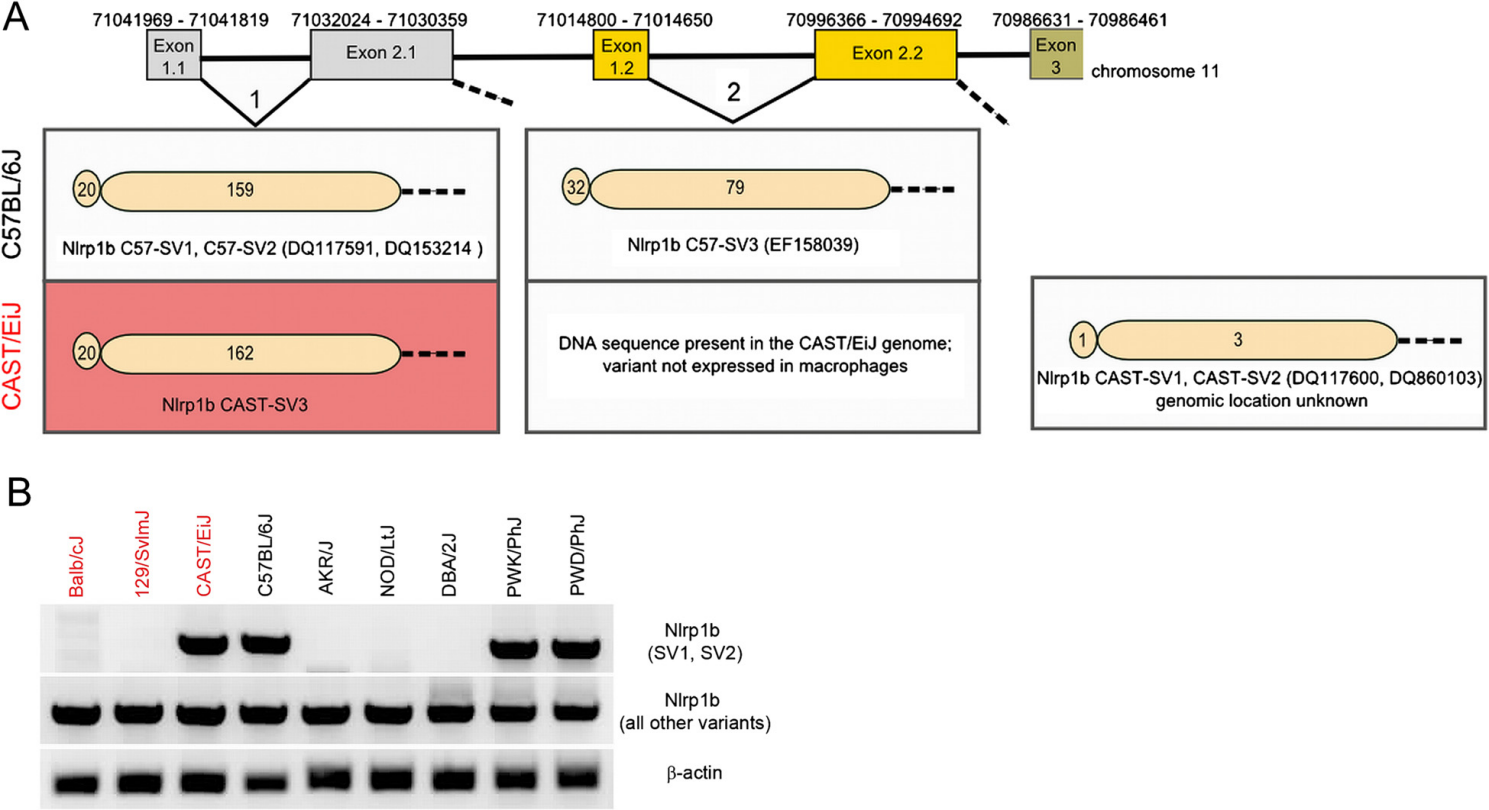
**(A) Organization of the *Nlrp1b *duplicated exons 1 and 2 on chromosome 11 of CAST/EiJ and C57BL/6J mice and model of alternative splices*.*** Arrangement of the duplicated exon 1 and 2 pairs encoding for different Nlrp1b variants on mouse chromosome 11. We propose the occurrence of two alternative splice variations. Splice event 1 (shown on left) results in a mature transcript encoding for *Nlrp1b* splice variant (SV) 1 and 2 in C57BL/6J and for SV3 in CAST/EiJ macrophages (in red). Splice event 2 (shown on right) results in a transcript encoding for *Nlrp1b* C57BL/6J SV3. In the CAST/EiJ genome, the SV3 sequence is present at this genomic location. The exact locations of the variants on chromosome 11 and GenBank accession numbers of described protein variants are given. Polymorphisms present in the variant proteins in comparison to the 129S1/SvlmJ protein sequence as outlined in Figure 3A are indicated by numbers. **(B)** Presence of *Nlrp1b* C57BL/6J splice variant (SV) 1 in the genome of diverse mice. The presence of exon 2 of *Nlrp1b* SV1 in the genome of mice harboring LT resistant (black) or sensitive (red) macrophages was analyzed. The upper panel shows the amplicons generated when specific primers were used to amplify *Nlrp1b* C57-SV1 or SV2, the middle panel shows genomic presence of sequences for all other variants, or of beta actin (control).

Prior to this study, it was assumed that *Nlrp1b* “alleles” of different mouse strains all shared the same genomic location. The finding that CAST/EiJ mice express two distinctively different *Nlrp1b* SVs (CAST-SV3 resembling C57-SV1, and the CAST-SV1/SV2) led us to more carefully examine the publically available *Nlrp1b* genomic sequence data to identify the exact location of the exons encoding for these variants. We used the genomic coordinates for exons 1 and 2 of the *Nlrp1b* C57-SV1 (CCDS 36210) and C57-SV3 (CCDS 48839) reported at the NCBI Consensus Coding sequence (CCDS) database (http://www.ncbi.nlm.nih.gov/projects/CCDS/CcdsBrowse.cgi) (assembly MGSCv37) to analyze the corresponding loci for exon 1 and 2 in the genome sequence of CAST/EiJ, available at Sanger (http://www.sanger.ac.uk). We found that exons 1 and 2 of C57-SV1 and C57-SV3 are duplicated, leading to a tandem arrangement of these pairs on the genome (Figure 4A). Furthermore, the genomic locus of our newly identified *Nlrp1b* CAST-SV3 was identical to that of the annotated C57-SV1 (Figure 4A). Additionally, we found that CAST/EiJ mice also harbor sequences identical to exon 1 and 2 of the C57-SV3 in their genome, at the same locus as C57BL/6J mice (Figure 4A). This *Nlrp1b* SV is apparently not expressed in macrophages of CAST/EiJ mice since all expression screens performed to date have failed to identify it. Nevertheless, these findings raised the question of the genomic location of the exons encoding for the *Nlrp1b* CAST-SV1 and SV2 sequences. However, although we downloaded CAST/EiJ chromosome 11 FASTA sequence from the Sanger site and performed a Blast search [20] for exon 2 of *Nlrp1b* CAST-SV1, we were unable to identify its location, which is likely due to the not yet completed sequencing in the *Nlrp1b* region of the CAST/EiJ genome (http://www.Sanger.ac.uk). These results show that CAST/EiJ mice have the genetic information to make at least 3 different *Nlrp1b* splice variants. This observation may open up new avenues for the investigation of LT-sensitivity in CAST/EiJ mice, which have also been shown to have some unique characteristics in LT intoxication studies [21].

To investigate the possibility that in addition to CAST/EiJ, other mice also harbor C57-SV1-like sequences in their genomes, we used genomic DNA as PCR template and our *Nlrp1b* C57-SV1 specific primers (Table 1) to screen for the presence of exon 2. However, we generated amplicons only for mice that had been proven positive in our previous expression screen (Figure 4B), indicating that either the tested mouse strains do not harbor these sequences, or that they contain polymorphisms, thus escaping recognition by our primers.

**Table 1:** Primers used in this study

**Name**	**5**^**′ **^**– 3**^**′**^** sequence**	**Purpose**
Nlrp1a-1F	CAGAAGAGCGTCTTGAAGCAA	Sequencing of *Nlrp1a*
Nlrp1a-2F	AAGCTTTACAGGAATGACTTCCAT	
Nlrp1a-3F	CCAGCTCAGAGACCTCTGCT	
Nlrp1a-4F	TTCAGCTGAACAGGAAGGTACAG	
Nlrp1a-5F	GGATAAAGAAGAAGTGGGTGATAGC	
Nlrp1a-6F	CTCCAAGAGGGAATCGTGG	
Nlrp1a-7F	CTGCCCAAGATTGCTACAGC	
Nlrp1a-1R	GAAGTCATTCCTGTAAAGCTTGC	
Nlrp1a-2R	AGGGTCCTTCTTTGGCAGAC	
Nlrp1a-3R	CCGTAAACTTATGTCCCTGCTG	
Nlrp1a-4R	TCATCTTCAGTCCCCAGAGG	
Nlrp1a-5R	AGTACTGCGTAATGCTGTTCCAC	
Nlrp1a-6R	GCTTGTCCAAGAGAGGATCTACAG	
Nlrp1a-7R	GTGCTCACTTTTCAGCCACC	
Nlrp1a-F	ACAGACATGGACCTCATGGTGGTT	Survey of *Nlrp1a* expression – 201 bp [17]
Nlrp1a-R	CAACTCCTCCAGGTTTCTGGCTAAC	
Nlrp1b_2_-F	ATGTGATGGTATGGAATTCAAAAGAACTG	Survey of *Nlrp1b* (SV1, SV2) expression – 503 bp
Nlrp1b_2_-R	AACCATACTGGGCAACTGTCAGCTT	
Nlrp1b_1_-F	ACACGGTAATATGGAAATGGACAGAATTG	Survey of expression of all other *Nlrp1b* variants– 487 bp
Nlrp1b_1_-R	AACCATCCTTGGGGATATCAGTGC	
Nlrp1c-FW	GACAAGGGCAGTGACAATTGAGATT	Survey of *Nlrp1c*
Nlrp1c-RV	ACATTTGGGGTCCTCAGTGTCACT	expression – 451 bp
Actin-F	TACAGCTTCACCACCACAGC	beta-actin control in genomic DNA – 206 bp
Actin-R	AAGGAAGGCTGGAAAAGAGC	

### Expression profile of *Nlrp1c*

**Figure 5:**
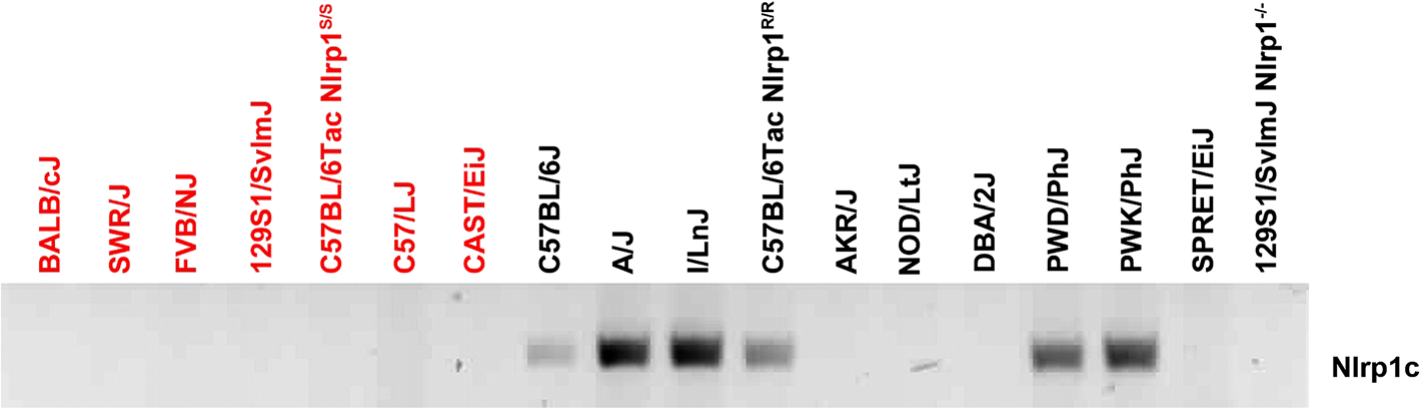
**Expression profile of *Nlrp1c.*** LT-resistant (black) or sensitive (red) macrophages of indicated mouse strains were PCR analyzed for expression of *Nlrp1c* using cDNA as template.

Analysis of *Nlrp1c* expression with previously published primers [17] revealed a 100% correlation with *Nlrp1a* expression data (data not shown). This intriguing observation and the high DNA sequence homology between the paralogs prompted us to sequence the presumed *Nlrp1c* amplicons produced from macrophages of C57BL/6J, NOD/LtJ, DBA/J, and CAST/EiJ mice. Surprisingly, only the C57BL/6J-derived amplicon had a 100% match to *Nlrp1c,* while the PCR fragments obtained from cDNA of all other mice had sequences exactly matching *Nlrp1a.* Therefore, we designed a new reverse primer based on an insertion sequence uniquely occurring in the 3-prime end of *Nlrp1c*, immediately after the last codon of exon 8 (Table 1), and used it in combination with the published forward primer. With these primers, only certain LT-resistant macrophages, mainly derived from mice known to harbor an *Nlrp1b* C57-SV1 and from the wild-derived mice PWD/PhJ and PWK/PhJ, expressed *Nlrp1c* (Figure 5). These results imply that when *Nlrp1c* is expressed, the previously published primers amplify it; however, in the absence of *Nlrp1c*, they amplify *Nlrp1a*. Our results indicate that *Nlrp1c* is expressed only in macrophages of LT-resistant C57BL/6J, A/J, I/LnJ, PWD/PhJ, and PWK/PhJ mice.

## Discussion

It has been known for years that macrophages from certain inbred mouse strains are rapidly lysed by anthrax LT, while macrophages from other strains are resistant [22]. In recent years it has been demonstrated that this cell death is mediated by activation of the Nlrp1b inflammasome [17]. However, mice contain three highly homologous, tandemly-arranged *Nlrp1* gene paralogs, which presumably arose by gene duplication, perhaps to provide an increased ability to respond to diverse stimuli. To date, there is limited information on expression of these paralogs in mice, nor on how the potential interactions of the resulting proteins might affect the response of mice to LT and other stimuli. We initiated an expression survey of all three paralogs in a set of inbred mice with the intent to elucidate whether the paralog proteins could act in a competitive manner in impacting sensitivity to LT-induced, Nlrp1b-mediated macrophage pyroptosis. The idea that the products of genes arising from duplication may acquire novel functions [23] and that they can modulate the activity of their paralogs is not novel. For example, in plants, the products of many of the R genes controlling responses to different pathogen effector proteins appear to have arisen by gene duplication [24]. Similarly, the mammalian protein cFLIP, which is a caspase-8 paralog lacking enzymatic activity, modulates procaspase-8 activation by heterodimerization [25,26]. Nlrp1a and Nlrp1c share over 70% protein sequence homology with Nlrp1b, and may thus act by competitively binding effector proteins, or by forming heterodimers with Nlrp1b, resulting in modulation of inflammasome activation. Thus, we investigated expression of all three paralogs in LT-resistant and sensitive macrophages from a large set of mice.

Our analysis of *Nlrp1a* expression showed a near-perfect correlation between toxin sensitivity and expression; however, macrophages of the LT-sensitive CAST/EiJ mice also expressed *Nlrp1a*, and its protein sequence was nearly identical to that of resistant mice, as determined by sequence analysis. Like most Nlrp1b proteins, Nlrp1a contains all domains necessary for inflammasome activation. To date, no danger signal resulting in activation of the Nlrp1a inflammasome has been described. However, it was recently shown that the transcription factor SREBP-1 (for sterol regulatory element binding protein-1), which has been linked to the regulation of cellular lipid levels, activates gene expression of *Nlrp1a* and/or *Nlrp1c*, likely by binding directly to promoter regions, but not of *Nlrp1b*[27]. The fact that *Nlrp1a* expression can be influenced by a cellular regulator implies that it may have importance as an intracellular sensor. Since in our study nearly all macrophages sensitive to LT did not express *Nlrp1a*, it may be interesting to investigate whether SREBP-1 can activate *Nlrp1a* gene expression in these macrophages, and whether this may change their sensitivity to LT. Furthermore, the outlier strain expressing *Nlrp1a* (CAST/EiJ) has been shown to have an interesting and rapid early response to LT in whole animal challenges with the toxin, and it is tempting to speculate on a role for Nlrp1a in that response [21]. This strain is also highly susceptible to LT relative to all other inbred strains [28], and it has been shown to have a deficient interferon gamma response during viral infection [29]. Thus, it appears that besides the expression of multiple *Nlrp1b* splice variants, CAST/EiJ mice have many more unique characteristics. Also intriguing is recent data that show *Nlrp1a* and *Nlrp1b* expression in resting neutrophils [30], which could have an additional impact on animal susceptibility to infection [19].

Perhaps the most interesting result from comparison of Nlrp1a sequences of different inbred strains was the discovery of the extreme conservation. In light of the fact that the closest gene to *Nlrp1a* in the mouse genome is *Nlrp1b*, it is remarkable that Nlrp1a is highly conserved in all analyzed strains whereas Nlrp1b appears to be highly polymorphic. These findings are likely indicative of independent evolutionary pressures on each gene, in the form of the danger signals they recognize or sense. In the case of Nlrp1b, it is interesting to speculate that the evolutionary pressure may be exposure to anthrax infection. The activator and function of Nlrp1a remains to be determined. Interestingly, murine Nlrp1a is more closely related to the only expressed rat Nlrp1 orthologs than to murine Nlrp1b or Nlrp1c [15]. The conservation of Nlrp1a suggests an important role for this gene, perhaps when expressed in other cell types. Our expression analysis showed lack of *Nlrp1a* expression in tissues of BALB/cJ mice; however, it cannot be excluded that gene expression is induced only in response to the (yet unknown) Nlrp1a danger signal.

When surveying the sequences for *Nlrp1b* deposited at GB, we discovered the presence of splice variants that have not been described and characterized before. The presence of these alternative splice variants in macrophages is of particular interest, as alternative splicing could occur in a tissue-specific manner, resulting in an increased variety of proteins depending on the cellular environment. It has been suggested that 90-95% of human pre-mRNAs from multi-exon genes can undergo alternative splicing [31,32]. To receive a complete assessment of *Nlrp1b* genes expressed in LT-sensitive and resistant macrophages, we designed primers that allowed for the differentiation between splice variants. We then surveyed expression of *Nlrp1b* and found it to be expressed in macrophages of all strains, excluding the *Nlrp1*^−/−^ control and the SPRET/EiJ mouse (*Mus spretus)*, the latter being a wild-derived mouse closely related to *Mus musculus*. The GB sequence for *Nlrp1b* of this mouse was named “allele 6” and it resembles the translated protein of C57-SV1 with 83% identity. Unlike the *Mus musculus* Nlrp1b variants which are highly conserved in the C-terminus, the Nlrp1b protein expressed by SPRET/EiJ shows many polymorphisms in this part of the protein. Because of sequence differences, it is likely that our primers were not specific enough for the amplification of this variant.

A surprising finding in our *Nlrp1b* expression screen was the fact that LT-sensitive macrophages of CAST/EiJ mice appeared to express at least two splice variants of which one appeared identical to the C57-SV1 *Nlrp1b* expressed by LT-resistant macrophages. These results indicate that in some mice, genes encoding for two Nlrp1b protein variants (or at least parts thereof) with opposing LT sensitivities can be expressed at the same time. Also, with the discovery of this additional splice variant (CAST-SV3) in CAST/EiJ macrophages, it becomes apparent that the previous annotation of *Nlrp1b* SVs as “alleles” is misleading, since *Nlrp1b* “alleles” of different mouse strains do not share the same genomic location. We hope that our new annotation of (splice) variants will not only allow to more clearly distinguish between true alleles and transcript variations thereof, but to also accommodate potential new splice variants that may be discovered in macrophages or in other cell types.

The paralog *Nlrp1c* is different from *Nlrp1b* and *Nlrp1a* since it does not encode for a full-length inflammasome sensor, but it is truncated after exon 8. We nevertheless included this likely inactive inflammasome sensor in our study since we could not exclude that although it is truncated, it could still interact with and compete for yet unidentified N-terminal Nlrp1b inflammasome binding partners. In contrast to a previous *Nlrp1c* expression screen in mouse macrophages, we did not observe expression of this paralog in macrophages of mice belonging to the groups defined by Boyden and Dietrich as allelic groups 3 (AKR/J and NOD/LtJ), 4 (DBA/J), and 5 (CAST/EiJ) [17]. We were able to explain this discrepancy by showing that primers used in the first study were non-specifically amplifying *Nlrp1a* in the absence of *Nlrp1c* expression. This discovery accentuates the necessity to carefully compare paralog sequences when analyzing gene expression. Nevertheless, our results indicate that macrophages of only a few mouse strains express this paralog, and that there is no obvious correlation between macrophage sensitivity to LT and its expression.

Although our survey did not allow us to establish a direct correlation between *Nlrp1* paralog expression and macrophage sensitivity to LT, it gave us important information on the presence of paralog transcripts and highlights the complexity of *Nlrp1* expression in mice. A summary of our results is given in Table 2.

**Table 2:** **Expression profile of *Nlrp1a*, *Nlrp1b *(splice) variants, and *Nlrp1c***

**Mouse strain**	***Nlrp1a***	***Nlrp1b *(SV1, SV2)**	***Nlrp1b *(all other variants)**	***Nlrp1c***
**BALB/cJ**	-	-	+	-
**SWR/J**	-	-	+	-
**FVB/J**	-	-	+	-
**129S1/SvlmJ**	-	-	+	-
**C57BL/6Tac Nlrp**^**S/S**^	-	-	+	-
**C57/LJ**	-	-	+	-
**CAST/EiJ**	+	+	+	-
C57BL/6J	+	+	+	+
A/J	+	+	+	+
I/LnJ	+	+	+	+
C57BL/6Tac Nlrp^R/R^	+	+	+	+
AKR/J	+	-	+	-
NOD/LtJ	+	-	+	-
DBA/2J	+	-	+	-
PWD/PhJ	+	+	+	+
PWK/PhJ	+	+	+	+
SPRET/EiJ	+	-	-	-
129S1/SvmlJ Nlrp^−/−^	-	-	-	-

Mouse strain names written in bold letters indicate that they harbor lethal toxin sensitive macrophages. +/− refers to presence or absence of PCR product.

## Conclusions

Our results indicate that the presence of multiple *Nlrp1b* splice variants in different inbred mice complicates the investigation of Nlrp1b-dependent responses to LT, to infection with *B. anthracis,* and perhaps more importantly, to yet-to-be-identified other stimuli. Of particular interest, the high degree of conservation of Nlrp1a compared to Nlrp1b suggests the importance of Nlrp1a as a likely functional sensor of a more common danger signal capable of exerting a more consistent evolutionary pressure. The *Nlrp1* paralogs may have arisen by gene duplication, but they likely have evolved to sense different danger signals.

## Methods

### Ethics statement

All animal experiments were performed in accordance with guidelines from the NIH and the Animal Welfare Act and were approved by the Animal Care and Use Committee at the National Institute of Allergy and Infectious Diseases, National Institutes of Health.

### Mice

Unless otherwise indicated, mice were purchased from Jackson Laboratories (Bar Harbor, Maine). Mice harboring LT-sensitive macrophages used in this study were 129S1/SvlmJ, Balb/cJ, FVB/NJ, SWR/J, CAST/EiJ, and C57/LJ. Mice with LT-resistant macrophages were C57BL/6J, A/J, I/LnJ, SPRET/EiJ, PWK/PhJ, PWD/PhJ, AKR/J, NOD/LtJ, and DBA/2J. The congenic C57BL/6Tac Nlrp1^S/S^ and Nlrp1^R/R^ mice were previously described [19]. Nlrp1 knockout mice having a 129S1/SvlmJ background were created by one of the authors of this manuscript (SL Masters) and are described in [18].

### Cell culture

L929 mouse fibroblasts which secrete macrophage colony-stimulating factor needed for monocyte proliferation, were cultured at 37°C and 5% CO_2_ in complete Dulbecco’s modified Eagles’s medium (complete DMEM) containing 10% fetal bovine serum, 10 mM HEPES, and 50 μg/ml gentamicin (all from Invitrogen, Carlsbad, CA). Bone marrow cells were isolated from mice and cultured in two thirds complete DMEM and one third L929-conditioned culture supernatant for 7 days with medium changes every 2–3 days.

### RNA isolation, cDNA synthesis, and PCR

RNA was isolated from bone marrow-derived macrophages (BMDMs) or from organ tissues by Trizol extraction as per the manufacturer’s protocol (Invitrogen), followed by DNaseI treatment. Synthesis of cDNA was performed with the Superscript III First Strand Synthesis System (Invitrogen). One μl of cDNA or 100–200 ng genomic DNA isolated from mouse tissue using the Qiagen DNeasy Blood and Tissue Kit (Qiagen, Valencia, CA) was used as template for PCR reactions with Illustra PuRe Taq PCR beads (GE Healthcare, Piscataway, NJ). The cycling conditions were the following: 95°C for 1 min, 40 cycles of 95° 30 sec, 58° 30 sec, 72° 60 sec, and 72° for 10 min. All primers are listed in Table 1. For *Nlrp1c* expression analysis, the designed reverse primer anneals to a sequence located immediately downstream of the last coding exon (exon 8).

### GenBank accession numbers

The DNA and translated protein sequences of all 5 Nlrp1a variants were deposited with GenBank and can be accessed under the following numbers: KC539856 (A/J); KC539857 (AKR/J); KC539858 (CAST/EiJ); KC539859 (DBA/2J); KC539860 (I/LnJ); KC539861 (PWK/PhJ).

### Data analyses

Protein and DNA sequence alignments were performed with MegAlign using ClustalW, sequencing data was analyzed using Seqman, and reverse complement analyses and protein translations were performed with EditSeq (all from Lasergene DNA Star version 8). Sanger sequence data for different mouse strains was retrieved from ftp://ftp-mouse.sanger.ac.uk/current_denovo, and exonic sequences were analyzed using the LookSeq site at the Sanger Center. Blast searches were performed using the blast program available at http://blast.ncbi.nlm.nih.gov.

## Competing interest

The authors declare that they have no competing interests.

## Authors’ contributions

SHL, MM, and IS conceived the study. IS and MM designed the experiments. IS and DC conducted the experiments. AM performed BLAST analyses. SLM generated Nlrp1−/− mice. IS, MM, and SHL wrote the manuscript. All authors read and approved the final manuscript.

## Supplementary Material

Additional file 1: Table S1 Tissue expression of *Nlrp1a* and *Nlrp1b* in mouse organs determined by end-point PCR. Click here for file

Additional file 2: Figure S1 Protein sequence alignment of the two deposited Nlrp1a sequences. Accession numbers are shown on the left and colors indicate different domains: green, nucleotide-binding domain; yellow, leucine-rich repeats; blue, function-to-find domain; red, caspase recruitment domain. Grey color shows differences between the two deposited sequences. Click here for file
