# Supplementary material for: Transcriptional analysis of the three Nlrp1 paralogs in mice
Source: BMC Genomics. 2013 Mar 18;14:188. doi: 10.1186/1471-2164-14-188 (PMC3641005; doi:10.1186/1471-2164-14-188)
Supplement: Additional file 1: Table S1 — Tissue expression of Nlrp1a and Nlrp1b in mouse organs determined by end-point PCR. [file 1471-2164-14-188-S1.pdf]

Additonal Table 1

Tissue expression of Nlrp1a and Nlrp1b in mouse organs determined by end-point PCR

	**Nlrp1a**		**Nlrp1b**		
**Splice variant**			**SV1, SV2**	**SV3**	
**Mouse strain**	**Balb/cJ**	**C57BL/6J**	**C57BL/6J**	**Balb/cJ**	**C57BL/6J**
Bone marrow	-	+	+	+	+
Spleen	-	+	+	+	+
Liver	-	+	+	+	+
Kidney	-	+	+	+	+
Heart	-	+	+	+	+
Lung	-	+	+	+	+
Brain	-	+	+	+	+
Stomach	-	IC	+	-	+
Muscle	-	+	+	+	+
Spinal Cord	-	+	+	+	+
Thymus	-	+	+	+	+
Adrenals	-	+	+	+	+
Uterus	-	IC	-	+	+
Ovaries	-	+	+	+	+

IC, inconclusive results; +/- refers to presence of absence of PCR product.
