# Supplementary figures and images for: Transcriptional analysis of the three Nlrp1 paralogs in mice
Source: BMC Genomics. 2013 Mar 18;14:188. doi: 10.1186/1471-2164-14-188 (PMC3641005; doi:10.1186/1471-2164-14-188)

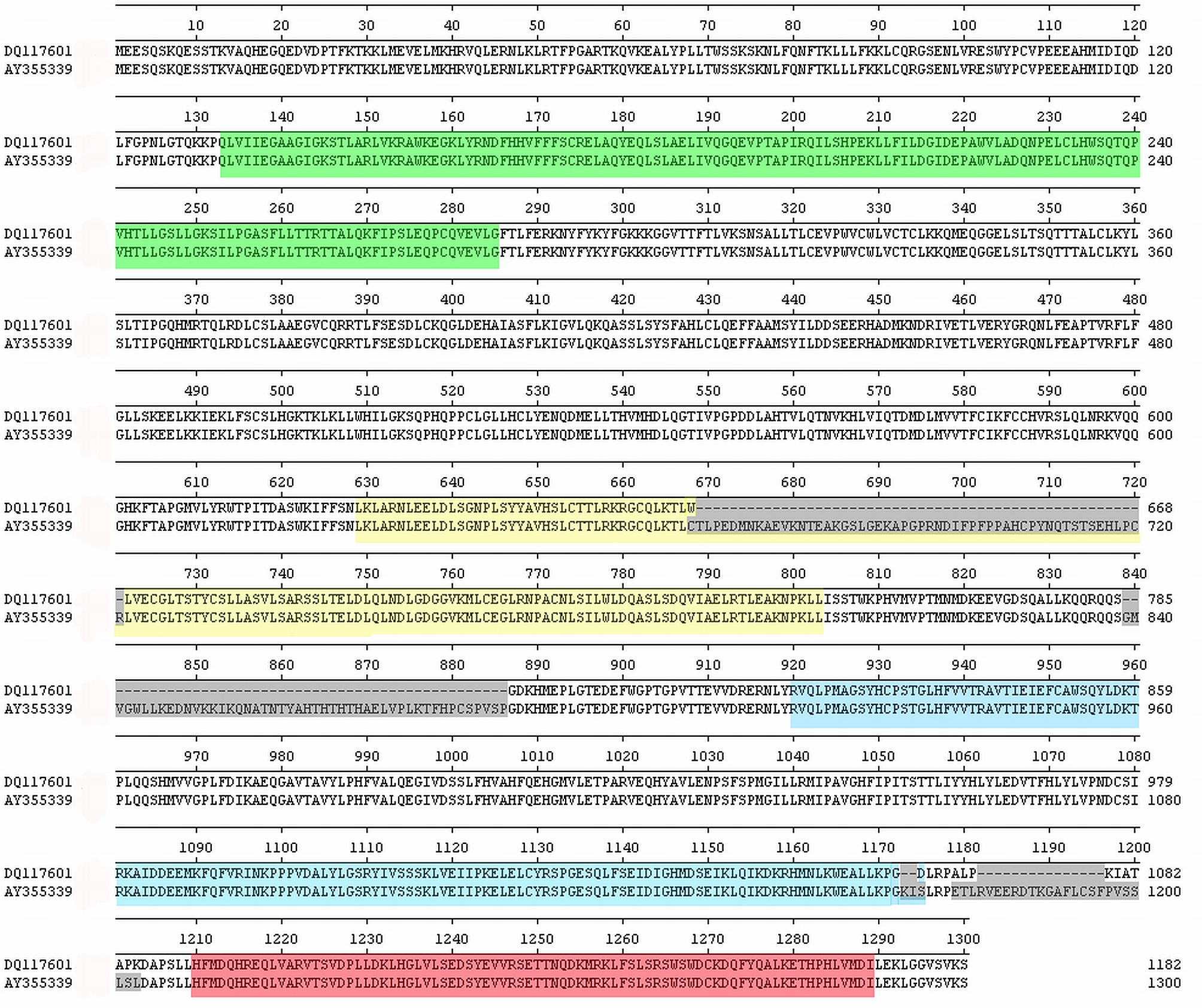

Supplement: Additional file 2: Figure S1 — Protein sequence alignment of the two deposited Nlrp1a sequences. Accession numbers are shown on the left and colors indicate different domains: green, nucleotide-binding domain; yellow, leucine-rich repeats; blue, function-to-find domain; red, caspase recruitment domain. Grey color shows differences between the two deposited sequences. [file 1471-2164-14-188-S2.jpeg]
